# Maturity Assessment of District Health Information System Version 2 Implementation in Ethiopia: Current Status and Improvement Pathways

**DOI:** 10.2196/50375

**Published:** 2024-07-26

**Authors:** Tesfahun Melese Yilma, Asefa Taddese, Adane Mamuye, Berhanu Fikadie Endehabtu, Yibeltal Alemayehu, Asaye Senay, Dawit Daka, Loko Abraham, Rabeal Tadesse, Gemechis Melkamu, Naod Wendrad, Oli Kaba, Mesoud Mohammed, Wubshet Denboba, Dawit Birhan, Amanuel Biru, Binyam Tilahun

**Affiliations:** 1 Department of Health Informatics Center for Digital Health and Implementation Science University of Gondar Gondar Ethiopia; 2 Department of Biostatistics and Epidemiology Center for Digital Health and Implementation Science University of Gondar Gondar Ethiopia; 3 Department of Computer Science Center for Digital Health and Implementation Science University of Gondar Gondar Ethiopia; 4 Department of Health Policy and Management Jimma University Jimma Ethiopia; 5 Digital Health Activity Addis Ababa Ethiopia; 6 Ministry of Health Addis Ababa Ethiopia; 7 Data Use Partnership Addis Ababa Ethiopia

**Keywords:** health information system, digital health system, District Health Information System version 2, DHIS2, maturity assessment, Stages of Continuous Improvement, Ethiopia

## Abstract

**Background:**

Although Ethiopia has made remarkable progress in the uptake of the District Health Information System version 2 (DHIS2) for national aggregate data reporting, there has been no comprehensive assessment of the maturity level of the system.

**Objective:**

This study aims to assess the maturity level of DHIS2 implementation in Ethiopia and propose a road map that could guide the progress toward a higher level of maturity. We also aim to assess the current maturity status, implementation gaps, and future directions of DHIS2 implementation in Ethiopia. The assessment focused on digital health system governance, skilled human resources, information and communication technology (ICT) infrastructure, interoperability, and data quality and use.

**Methods:**

A collaborative assessment was conducted with the engagement of key stakeholders through consultative workshops using the Stages of Continuous Improvement tool to measure maturity levels in 5 core domains, 13 components, and 39 subcomponents. A 5-point scale (1=emerging, 2=repeatable, 3=defined, 4=managed, and 5=optimized) was used to measure the DHIS2 implementation maturity level.

**Results:**

The national DHIS2 implementation’s maturity level is currently at the defined stage (score=2.81) and planned to move to the manageable stage (score=4.09) by 2025. The domain-wise maturity score indicated that except for ICT infrastructure, which is at the repeatable stage (score=2.14), the remaining 4 domains are at the defined stage (score=3). The development of a standardized and basic DHIS2 process at the national level, the development of a 10-year strategic plan to guide the implementation of digital health systems including DHIS2, and the presence of the required competencies at the facility level to accomplish specific DHIS2-related tasks are the major strength of the Ministry of Health of Ethiopia so far. The lack of workforce competency guidelines to support the implementation of DHIS2; the unavailability of core competencies (knowledge, skills, and abilities) required to accomplish DHIS2 tasks at all levels of the health system; and ICT infrastructures such as communication network and internet connectivity at the district, zonal, and regional levels are the major hindrances to effective DHIS2 implementation in the country.

**Conclusions:**

On the basis of the Stages of Continuous Improvement maturity model toolkit, the implementation status of DHIS2 in Ethiopia is at the defined stage, with the ICT infrastructure domain being at the lowest stage as compared to the other 4 domains. By 2025, the maturity status is planned to move from the defined stage to the managed stage by improving the identified gaps. Various action points are suggested to address the identified gaps and reach the stated maturity level. The responsible body, necessary resources, and methods of verification required to reach the specified maturity level are also listed.

## Introduction

### Background

Health information systems (HISs) have become an essential component of evidence-based decision-making and health service delivery worldwide [1,2]. Over the past years, countries have recognized the importance of reliable health data to track key health issues and outcomes, leading to significant investments in HISs in both high- and low-income nations. Therefore, the global community has recognized that reliable health data are vital for the development of national health systems. This has been further highlighted in the 2030 Agenda of Sustainable Development Goals [3,4], which recognizes the potential of information and communication technology (ICT) to accelerate human progress, bridge the digital divide, and develop knowledge societies [5].

In low- and middle-income countries (LMICs), various national governments and some global partners, such as the World Health Organization (WHO) and United Nations International Children’s Emergency Fund, have made substantial investments in strengthening their HISs [6]. In particular, Ethiopia has made remarkable achievements in implementing HIS over the years. In 2006, the Ministry of Health (MOH) undertook a Health Management Information System reform with a focus on data management, human resources, and ICT (Planning and Programming Department, unpublished data, May 2006) [7]. The MOH standardized the indicators, recording and reporting forms, procedures, and reporting channels to improve performance [8].

Moreover, the MOH has implemented different digital health initiatives, including District Health Information System version 2 (DHIS2), electronic community HIS, electronic medical record systems, electronic community-based health insurance, logistic management information system, human resource information system, master facility registries, and electronic public health emergency management system. Maturity assessment of such digital health systems is essential to guide policy makers in strategizing and prioritizing initiatives [7].

Despite the remarkable progress made in Ethiopia, a comprehensive maturity assessment is yet to be conducted to determine the maturity level of the system and to inform policy makers about potential future interventions. DHIS2 is an open-source digital health platform developed by the University of Oslo in 2006 to manage HISs. Its first implementation was in India in 2006, and its first national rollout was in Kenya in 2010 [9]. Since 2010, LMICs worldwide have adopted this software. In Ethiopia, DHIS2 has been implemented since 2018 [7]. Since then, the MOH has made significant achievements in the development and implementation of DHIS2, such as the deployment of web-based or offline instances, full ownership of DHIS2 customization and implementation, upgrading DHIS2 to version 2.30 with apps developed in-house such as those for disease and public health emergency management report, data visualization applications (scorecard, bottleneck analysis, action tracker, and custom reports), creating metadata, data set customization, incorporating reporting functionality, and new features for decision-making by integrating data quality checks and dashboards [10].

Currently, the MOH uses DHIS2 for planning, reporting, analysis, and dissemination of data for all health programs. It accurately and timely collects and aggregates data such as routine health facility data, staffing, equipment, infrastructure, population estimates, disease outbreaks, survey or audit data, patient satisfaction surveys, and longitudinal patient records. DHIS2 stores, analyzes, and evaluates both aggregate and event-based data at a health facility level [7]. Therefore, DHIS2 is accepted as a primary source of information for planning and decision-making in Ethiopia’s health system. However, despite its implementation, the maturity level of DHIS2 has not yet been assessed. A maturity assessment is used to measure the current maturity status of a certain HIS to identify the strengths and improvement points and accordingly prioritize the next steps to reach higher maturity levels [11].

In Ethiopia, the Stages of Continuous Improvement (SOCI) tool was used to assess the current maturity status and improvement of the overall HIS. The assessment report indicated that the overall maturity level of the Ethiopian HIS is between the “repeatable” and “defined” maturity stages [12]. The report recommends maturity assessment of individual digital health systems (eg, DHIS2), which have a wide impact and coverage in the country.

### Objective

This study aims to assess the maturity level of DHIS2 implementation in Ethiopia and propose a road map that could guide the progress toward a higher level of maturity. The assessment could help identify the current capabilities; set a baseline for measuring improvement; identify strengths, weaknesses, opportunities, and threats; identify factors influencing achievements; set new pathways for improvements; and foster a culture of excellence in Ethiopia.

## Methods

### Assessment Setting

The maturity assessment of DHIS2 implementation was conducted in Ethiopia. It is a landlocked country in East Africa, with a population of 118 million and a 3-tier health care system consisting of primary health care units (health posts, health centers, and primary hospitals), general hospitals, and tertiary hospitals. The country has implemented DHIS2 since 2018. Since then, the country has made significant achievements in the customization and implementation of DHIS2 [10].

### Assessment Procedure

#### Overview

**Figure 1:**
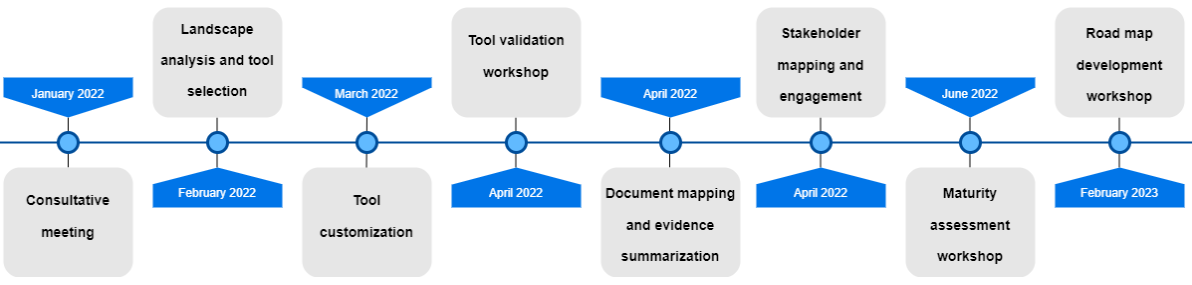
Diagrammatic representation of the assessment procedure.

A multimethod approach study was conducted in Ethiopia to assess the maturity level of the DHIS2 implementation. The assessment process proposed in the users’ guide for the HIS interoperability maturity assessment was followed to conduct the DHIS2 maturity assessment [13]. First, we conducted various consultative meetings to develop an activity plan for the maturity assessment. Then, we conducted a landscape analysis of various maturity assessment tools to select an appropriate tool for customization. Next, tool customization, followed by a validation workshop, was conducted. The purpose of the workshop was to validate the customized tool for its clarity and appropriateness in the Ethiopian context. Then, we mapped the stakeholders and conducted a maturity assessment workshop. Finally, a road map development workshop was conducted to improve DHIS2 implementation in Ethiopia. Figure 1 shows a diagrammatic representation of the assessment procedure.

#### Conducting Consultative Meetings

After the proposal to conduct a maturity assessment for DHIS2 was approved, the researchers conducted several consultative meetings with the MOH and Digital Health Activity, a partner of the MOH that supports Ethiopia in improving health care services through the implementation of digital health solutions. The first meeting was conducted on January 14, 2022, to discuss a drafted activity plan for the entire assessment procedure. The activity plan was presented in the presence of focal persons from the MOH and project facilitators from Digital Health Activity. Several useful inputs were provided to improve the activity plan. The key activities included a landscape analysis; a panel discussion among domain experts to select and customize the maturity assessment tool; a tool validation workshop involving domain experts, partners, and stakeholders to standardize the customized maturity assessment tool; a national workshop involving the MOH, partners, universities, and stakeholders; and data collection and analysis.

#### Landscape Analysis and Tool Selection

The researchers conducted a rigorous review of existing maturity assessment toolkits and peer reviewed publications from January 11, 2022, to February 15, 2022, to select an appropriate tool. Several tools were reviewed, including the Information Systems for Health Maturity Assessment Toolkit (IS4H) [14], the Global Digital Health Index [15], the Digital Maturity Assessment Tool [16], the HIS Interoperability Maturity Toolkit [17], the Hospital Information System Maturity Model [17], and the SOCI toolkit [18].

After reviewing each potential assessment model and conducting several discussion meetings, 2 models were shortlisted, considering their relevance, comprehensiveness, validity, credibility, and feasibility: IS4H and SOCI. IS4H is a maturity assessment model that describes the method, tool, and questions for assessing the organizational capacity of digital health systems. The model was developed by the Pan American Health Organization and WHO. In contrast, SOCI was developed by the US Centers for Disease Control and Prevention, the Health Data Collaborative Digital Health and Interoperability Working Group, and the United States Agency for International Development–funded Monitoring and Evaluation to Assess and Use Results Evaluation to help countries assess, plan, and prioritize interventions and investments to strengthen an HIS.

After rigorous deliberations regarding the strengths and limitations and comparing the shortlisted models (Multimedia Appendix 1) in a discussion among experts from the MOH, partners, and universities, SOCI was selected as the final model to assess DHIS2 implementation maturity.

#### Tool Customization

The assessment measures the current and desired HIS status across 5 core domains, 13 components, and 39 subcomponents and road map improvements [18]. The assessment tool measures maturity level based on 5 stages—emerging, defined, repeatable, managed, and optimized, with scores ranging from 1 to 5. Table 1 shows the definitions of each stage of the SOCI tool.

The tool was designed in 2 formats: an Excel (Microsoft Corp)–based and an app-based version of the tool available through the DHIS2 platform. In our case, we customized the Excel-based tool in March 2022 to assess the DHIS2 implementation maturity status. The customization focused on changing the generic HIS to the DHIS2 context. Throughout the Excel-based tool, we have modified the HIS concept to the DHIS2 context without altering the purpose and the core domains, components, and subcomponents of SOCI.

**Table 1:** The 5 stages of the Stages of Continuous Improvement tool and their definitions.

Stage	Scale	Definition
Emerging	1	Formal processes, capabilities, experience, or understanding of HIS^a^ issues or activities are limited or emerging. Formal processes are not documented, and functional capabilities are at the development stage. Success depends on individual effort.
Repeatable	2	Basic processes are in place, based on previous activities or existing and accessible policies. The need for standardized processes and automated functional capabilities is known. There are efforts to document the current processes.
Defined	3	There are approved documented processes and guidelines tailored to District HIS version 2 activities. There is increased collaboration and knowledge sharing. Innovative methods and tools can be implemented and used to extend the functional capabilities.
Managed	4	Activities are under control using established processes. Requirements or goals have been developed, and a feedback process is in place to ensure that they are met. Detailed measures for processes and products are being collected.
Optimized	5	Best practices are being applied, and the system is capable of learning and adapting. The system uses experiences and feedback to rectify problems and continuously improve processes and capabilities. Future challenges are anticipated, and a plan is in place to address them through innovation and new technology. Processes are in place to ensure reviews and incorporation of relevant innovation.

^a^HIS: health information system.

#### Tool Validation Workshop

A validation workshop involving 18 high-level digital health experts was conducted on April 28 and 29, 2022, to standardize and validate a customized SOCI tool, with participants forming 2 groups to validate each domain, component, and subcomponent and provide constructive feedback. Following the workshop, the feedback was incorporated, which resulted in a validated and standardized SOCI tool.

#### Document Mapping and Evidence Summarization

The assessors used evidence from the policies and guidelines available in the Ethiopian MOH resource library to evaluate the DHIS2 implementation maturity status [19]. This supporting evidence was mapped in April 2022 based on the content and topics related to the specific component, which served as a reference for the assessors.

#### Stakeholder Mapping and Engagement

Potential stakeholders were identified in April 2022 after a discussion with the MOH, and those who were working on and supporting DHIS2 were selected, including national and international partners and agencies such as Ethiopian Food and Drug Administration; Ethiopian Pharmaceuticals Supply Agency; Ethiopian Public Health Institute; United Nations International Children’s Emergency Fund; United States Agency for International Development; Water, Education, Economic Empowerment, Medical Care, and Alliance; Program for Appropriate Technology in Health; Bill and Melinda Gates Foundation; WHO; Population Services International; HIS Program; Institute of Chartered Accountant of Pakistan; African Medical and Research Foundation; Children’s Investment Fund Foundation; Last 10 Kilometers; Data Use Partnership; Transform Primary Health Care; Project Hope; and Clinton Health Access Initiative. All directorates under the MOH, regional health bureaus, and universities were officially invited to participate in the maturity assessment, and most of the selected stakeholders agreed to participate.

#### Maturity Assessment Workshop

A total of 35 digital health experts were invited to participate in the maturity assessment workshop, of which only 29 (83%) participated. The workshop was conducted from June 15, 2022, to June 17, 2022, with participants representing the stakeholders identified in the mapping exercise. The workshop began with an opening remark by the Health Information Technology Director stating the workshop’s objectives and the aim of the assessment, followed by a presentation on the basics, rationale, methods, and reasons for maturity assessment by the University of Gondar team. The maturity assessment tool selection process, landscape analysis, steps followed, and activities performed were presented. Participants were categorized into 2 major groups based on their expertise and experiences and oriented on how to conduct the assessment using the adopted maturity assessment toolkit, SOCI. Each team assessed DHIS2 based on the 5 domains of SOCI, with chairpersons and secretaries facilitating and documenting the assessment scores and justification for each result. Disagreements in scoring between the groups were resolved through discussions. The average score of the 39 subcomponents was summed as the total score for each component, the average score of the 13 components was summed as the total score for each domain, and the average score of the 5 domains was considered as the total score of the current DHIS2 implementation maturity. Finally, the assessment scores, strengths, and gaps in the DHIS2 implementation were presented and discussed, and feedback was incorporated to refine the assessment results.

#### Road Map Development Workshop

Of the 45 digital health experts invited to the road map workshop, only 38 (84%) participated. The workshop was conducted from January 30, 2023, to February 2, 2023. The workshop aimed to improve the maturity level of DHIS2 implementation. In total, 38 high-level digital health system experts—12 from the MOH, 9 from Regional Health Bureaus, 2 from agencies, 10 donors or partners, and 5 from Capacity Building and Mentorship Program Universities—participated. The workshop was hands-on, with at least 90% of the time spent in brainstorming and discussion. This approach allowed for better experience, more retention, and more realistic evaluation of the roadmap development. The sessions were continuously linked to the strategic challenges faced by the MOH or health sector, and solutions were discussed and suggested to provide practical solutions for DHIS2 maturity challenges.

During the workshop, key issues related to the 5 domains, 13 components, and 39 subcomponents of the SOCI tool were addressed for the road mapping of DHIS2 implementation. Various discussions were conducted to uncover the strengths and gaps of DHIS2 implementation; identify specific limitations that hinder the capability and implementation of the road map of DHIS2; analyze how DHIS2 is implemented; explore various ways of future improvements on DHIS2 including alternative concepts, tools, and technologies; and scrutinize various governance documents, tools, system documentation, manuals, and other resources to assess the maturity road map of DHIS2.

Each participant integrated knowledge, cognitive strategies, and behaviors as a guide in the group work, and the road map tool was presented and discussed. A detailed road map for each domain and respective components and subcomponents was presented, and major gaps and methods to address them were discussed. A 2-year target was set, and a list of high-impact interventions was identified to address the gaps. Considerations and means of verifications were established; the primarily responsible body was identified; and finally, the road map was developed.

### Ethical Considerations

The maturity assessment was conducted in a workshop where participants completed the assessment in groups. There was no individual assessment. No personal identification was used in the assessment. Participation was voluntary. Participants were informed to express their ideas freely during the group discussion.

### Implementation (Results)

#### Overall Current and Target Maturity Levels

**Figure 2:**
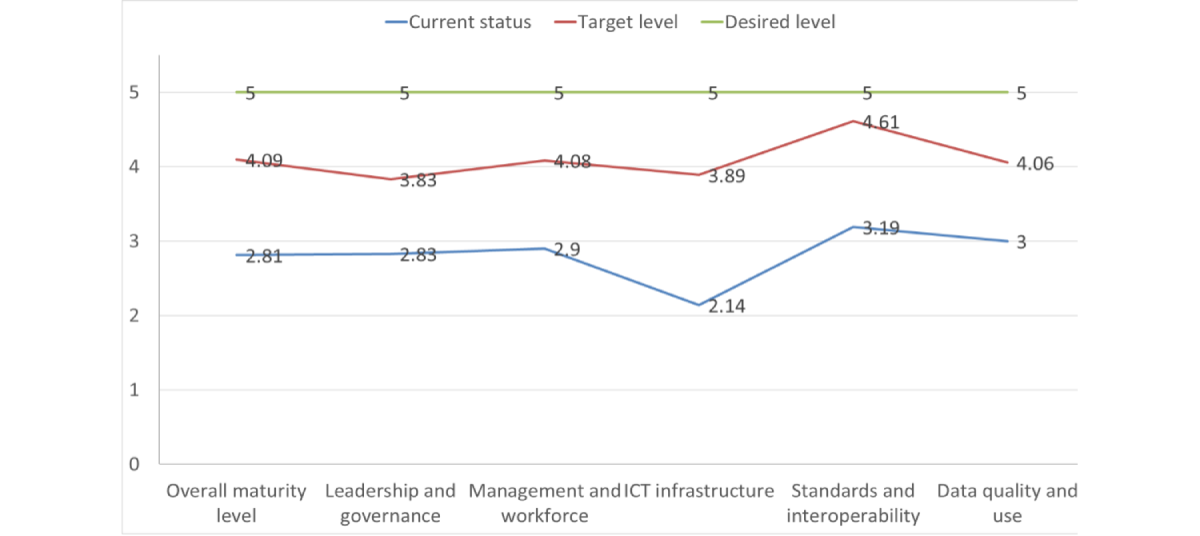
A line graph showing the overall national maturity levels and targets of District Health Information System version 2 implementation. ICT: information and communication technology.

The overall DHIS2 implementation in the country is currently at the “defined” stage, with a score of 2.81, and the goal is set to reach the “managed” stage of maturity, with a score of 4.09 by 2025 (Figure 2). Except for the ICT infrastructure domain, which is at the “repeatable” stage, with a score of 2.14, the other 4 domains are at the “defined” stage, with a score of approximately 3. The MOH’s strengths include the development of a standardized and basic DHIS2 process at the national level, the development of a 10-year strategic plan to guide the implementation of digital health systems, and the presence of required competencies at the facility level. However, the lack of workforce competency guidelines, the unavailability of the core competencies required to accomplish DHIS2 tasks at all levels of the health system, and ICT infrastructure challenges are the major hindrances to effectively implementing DHIS2. DHIS2 implementation is also in the “emerging” stage in terms of interoperability and data use components. Multimedia Appendix 2 provides the maturity levels of each domain, component, and subcomponent for the DHIS2 implementation.

#### Domain-Wise Implementation Status and Improvement Road Map

##### Leadership and Governance

**Figure 3:**
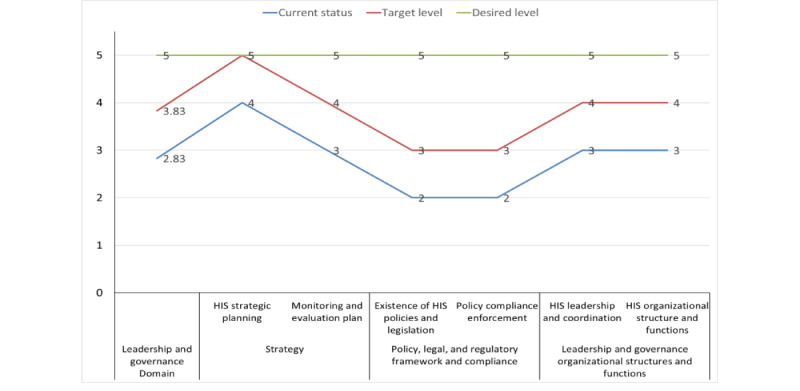
District Health Information System version 2 leadership and governance maturity level and targets. HIS: health information system.

The target maturity level for the DHIS2 leadership and governance domain is to move from the defined (score=2.83) to the managed (score=3.83) stage. The “strategic planning” component is intended to move from the managed (score=3.5) to the optimized (score=4.5) maturity stage. The “policy, legal, and regulatory framework, and the compliance” component is intended to progress from the repeatable (score=2) to the defined (score=3) maturity stage, whereas the “leadership, governance, organizational structures, and functions” component is intended to move from the defined (score=3) to the managed (score=4) maturity stage (Figure 3). The MOH has a well-defined and budgeted strategy for HIS that includes DHIS2. In addition, there is a national-level cross-agency coordination group that oversees DHIS2 implementation. However, there are no well-defined mechanisms and regulatory bodies to ensure adherence to organizational policies, procedures, and best practices related to the digital health system. Multimedia Appendix 3 provides detailed information about the strengths and gaps of the ministry regarding DHIS2 implementation with respect to the “leadership and governance” domain.

The bodies responsible for performing the suggested activities (Multimedia Appendix 3) for the subcomponents are the MOH, Ethiopian Food and Drug Administration, regional health bureaus, subregional health administration authorities, and all line agencies. The resources needed to perform the operations indicated for the leadership and governance domain are adequate time, sufficient number of competent workforces, and adequate funds.

##### Management and Workforce

**Figure 4:**
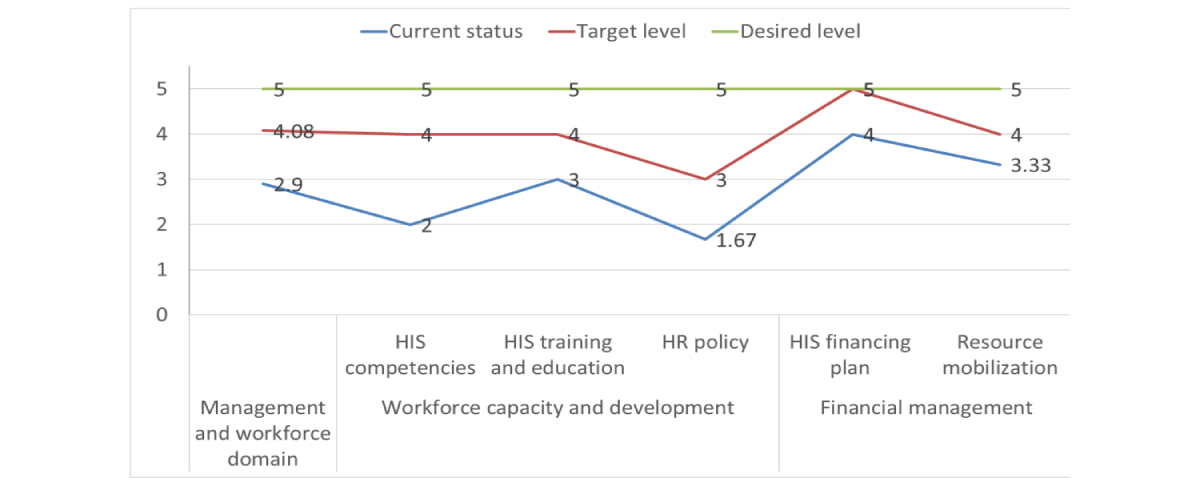
A line graph indicating the national District Health Information System version 2 management and workforce domain maturity level and targets. HIS: health information system; HR: human resource.

The DHIS2 “management and workforce” domain is targeted to improve from the defined (score=2.9) to the managed (score=4) maturity stage. Financial management and workforce capacity and development are the 2 components that are expected to advance from the managed (score=3.6) to the optimized (score=4.5) stage and the repeatable (score=2.2) to the managed (score=3.67) stage, respectively (Figure 4). The DHIS2 academy-level training, collaboration with institutions, and customized training are identified as promising workforce competency initiatives. However, irregular DHIS2 workforce capability assessments and analyses, unclear career paths and roles, insufficient infrastructure, lack of regular feedback, and unmet staffing needs were identified as challenges (Multimedia Appendix 4). The MOH, Human Resource Directorate, Regional Health Bureau, higher institutions, associations, and civil service are responsible for preforming the planned activities. Financial and human resources, infrastructure, and connectivity are required resources. Verification methods included competency assessments, finance and monitoring and evaluation reports, and improvement at the job evaluation grading level.

##### ICT Infrastructure

**Figure 5:**
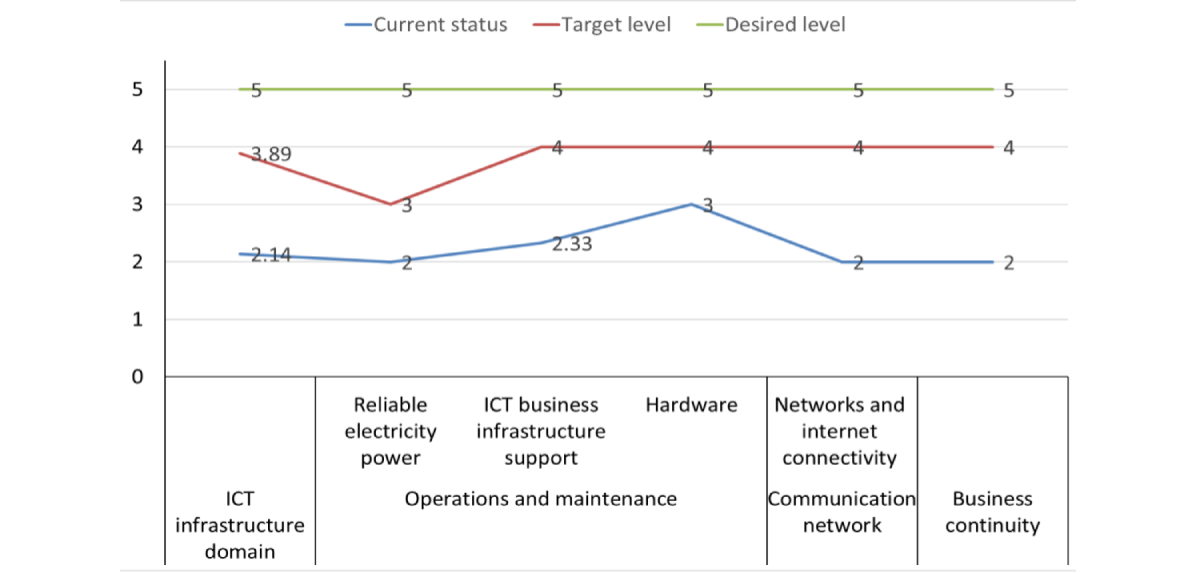
A line graph indicating the national District Health Information System version 2 information and communication technology (ICT) infrastructure maturity level and targets.

The information and communication domain’s projected maturity levels ranged from repeatable (score=2.14) to managed (score=3.89), with a plan in place to elevate operations and maintenance from the repeatable (score=2.44) to the managed (score=3.67) level. Upgrading the local area network and wide area network components from the repeatable (score=2) to the managed (score=4) level is also planned. Similarly, the business continuity element is slated for transformation from the repeatable (score=2) to the managed (score=4) level (Figure 5). The national MOH has collected data about electricity or power access, sources, and reliability to support DHIS2 infrastructure; however, it is limited at higher levels. The MOH has also developed the facility hardware and software specifications, with some national and subnational offices having adequate hardware. Maintenance and installation of DHIS2 infrastructure are currently handled through an ad hoc mechanism (ie, there is no regular maintenance and installation). Maintenance and installation are conducted when the need arises. Simultaneously, several challenges impede the effectiveness of DHIS2 implementation under this domain, such as unstable electricity, insufficient hardware, outdated infrastructure, and limited and unstable network and internet connectivity. In addition, business continuity plans are at the emerging stage, not harmonized across DHIS2 health sector needs, and infrequently reviewed and revised (Multimedia Appendix 5). The MOH, including the chief executive officer, Regional Health Bureaus, digital health, and ICT and health infrastructure departments, is responsible for executing the planned actions in the ICT infrastructure sector. Financial resources, electrical engineers, network specialists, maintenance professionals, ICT spare parts (eg, hard drives and RAM), and other ICT accessories are all essential for achieving the predetermined goals. The methods of verification in the ICT infrastructure field include evaluation reports, network assessments, and support requests, which are measured against predetermined indicators for support from the support system.

##### Standards and Interoperability

**Figure 6:**
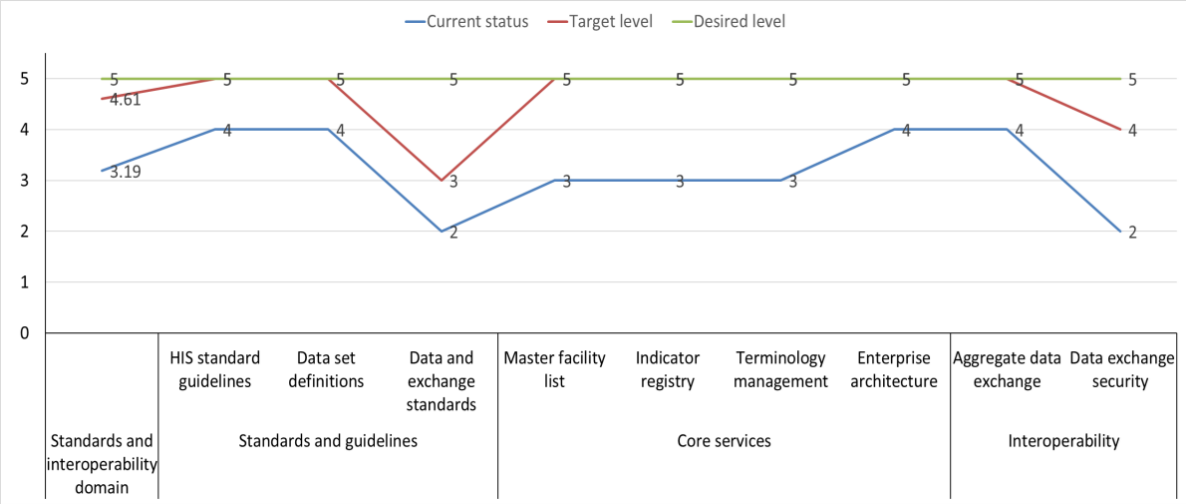
A line graph indicating the national District Health Information System version 2 interoperability and standard maturity level and targets.

The “standards and interoperability” domain is an indispensable element in ensuring the success of any HIS as it facilitates the exchange of data across various systems while ensuring the accuracy of data capture and reporting. The domain’s anticipated maturity levels ranged from defined (score=3.19) to optimized (score=4.61), with several components expected to move from the defined to the managed or optimized levels. The components of “standards and guidelines” are planned to have a maturity level ranging from defined (score=3.33) to managed (score=4.33), whereas the “core services” components are expected to move from the defined (score=3.25) to the optimized (score=5) level. In addition, there is a strategy to enhance the maturity level of the “interoperability and data exchange” components from defined (score=3) to optimized (score=4.5; Figure 6).

There are several strengths of the “standards and interoperability” domain, including the regular review and harmonization of indicators with international standards, the availability of different types of data exchange mechanisms, and well-documented indicator reference guides and formulas within DHIS2. The eHealth architecture includes an interoperability layer and defined shared services, and data exchange is piloted between DHIS2 and the Master Facility Registry. Vaccines, tracer drugs, and Rapid Diagnostic Test kits are available as indicators or data elements, and essential IT security measures such as virtual private networks, antivirus software, authentication, authorization, and firewalls are in place. Despite the ongoing efforts to improve standards and interoperability, there are still some gaps and areas for improvement. Although the national standard disease definitions, Health Management Information System recording and reporting guidelines, and electronic health record standards are in place, automated patient data exchange using internationally recognized standards between DHIS2 and other systems is not yet implemented. In addition, an interoperability laboratory for new exchange partners to test or onboard and a certification process do not exist yet. The DHIS2 data management and exchange standards are not integrated into the national health plan (Multimedia Appendix 6).

Technical expertise and financial resources are required to ensure the successful implementation and continued improvement in the “standards and interoperability” domain. The MOH and its partners are responsible for implementing the standards and interoperability-related activities. In addition, there must be a commitment to integrate DHIS2 data management and exchange standards into the national health plan and establish an interoperability laboratory for new exchange partners to test or onboard and a certification process.

##### Data Quality and Use

**Figure 7:**
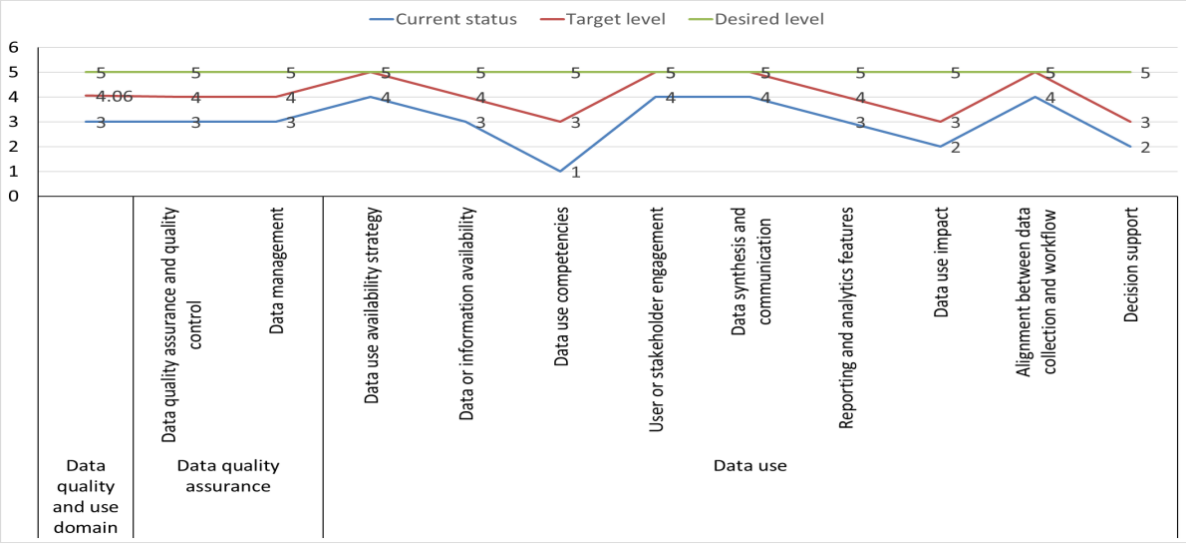
A line graph showing the national District Health Information System version 2 data quality and use maturity level and targets.

The anticipated maturity levels of the “data quality and use” domain ranged from defined (score=3) to managed (score=4.06), with planned improvements in the maturity level of data quality assurance components from the defined (score=3) to the managed (score=4) stage and data use components from the defined (score=3) to the managed (score=4.11) stage (Figure 7).

The assessment identified the strengths and gaps in this domain. The following strengths were identified: data quality assessment and auditing are performed regularly, manuals for data management and accessibility exist, and analytics and findings are shared with stakeholders on a quarterly basis. In addition, DHIS2 automated data reporting is implemented at all health facilities nationally, including sex-disaggregated data, where applicable. In contrast, there is no evidence of a national data quality governing body that meets regularly to ensure that data quality is maintained. Furthermore, the standard operating procedures and supervision for data management are inadequate. There is also a lack of common measurement metrics or indexes to monitor the progress of data or information use (Multimedia Appendix 7).

The means of verification for the “data quality and use” domain include documentation, data quality audit reports, regular technical working group minutes, practices of data exchange between systems, supervision reports, and regularly reviewed standard operating procedures. The MOH and its partners are responsible for implementing the standards- and interoperability-related activities in this domain. Technical expertise and financial resources are required to complete the tasks mentioned in the areas of standards and interoperability.

## Discussion

### Principal Findings

#### Overview

The maturity assessment aims to determine the current implementation status of DHIS2 and set a road map for improvement. The results show that the DHIS2 implementation status has an average score of 2.81, indicating that it is closer to the defined (stage 3) level. This suggests that there is a standardized and basic DHIS2 process at the national level. However, the implementation of DHIS2 is limited by the lack of guidelines for relevant workforce competencies. The competencies required for DHIS2 tasks, such as knowledge and skills, are not specific to DHIS2 activities. The biggest hindrance to DHIS2 implementation in Ethiopia is the ICT infrastructure, including network and internet connectivity. This finding is consistent with the HIS maturity assessments conducted by the MOH of Ethiopia [12] and WHO [20]. It is also consistent with other African countries, such as Cape Verde, Ghana, Mali, Nigeria, and Benin, where the HIS is at the “desired” level of maturity [20].

#### Leadership and Governance

According to this assessment, the “leadership and governance” domain is currently closer to the defined stage of maturity, with an average score of 2.83. This indicates that the monitoring and evaluation of DHIS2 implementation lacks a clear definition as well as adherence to organizational policies, procedures, and best practices related to the digital health system. This issue is not unique to Ethiopia, as it is also prevalent in many African countries [21,22]. Situational analysis of the Africa Health Strategy 2016 to 2030 revealed that most member states have poor HIS strategies [23]. Ethiopia has just started implementing strategies to facilitate the implementation of digital health systems including DHIS2 [24]. Furthermore, various studies conducted by organizations such as WHO and the Health Metrics Network have shown that health policy is one of the weakest components of digital health and HISs in many countries, particularly LMICs [25-31]. Weak leadership and insufficient coordination are major threats to HIS implementation in LMICs [32-34]. To address these challenges, strengthening the governance and regulation of technologies, including data privacy and security and accreditation of health apps for consumers, should be prioritized for good HIS governance and leadership [35]. A robust governance framework is essential to promote HIS accountability through monitoring and regular, transparent reviews of progress and performance (MOH, unpublished data, September 2022). By prioritizing these areas, Ethiopia should work toward achieving a more effective and sustainable DHIS2 system, ultimately leading to better health care outcomes for its populations.

#### Management and Workforce

Regarding the “management and workforce” domain, the assessment revealed that DHIS2 implementation in Ethiopia has reached the defined stage (score=2.9). This suggests that there is a lack of regular workforce capability assessments, unclear career paths, and insufficient infrastructure, hindering the effective management and workforce practice of DHIS2 functions at the national level. The importance of effective HIS management and workforce cannot be overstated, as it is crucial for evidence-based decision-making, health service planning, and the delivery of high-quality care [36,37]. Consequently, there has been a growing interest in developing a competent HIS workforce in Africa and beyond [35,38,39]. Financing is also vital for the maintenance, development, promotion, and expansion of DHIS2, necessitating the need for an integrated system of health collaborations and programs [38,40-43]. However, studies in Ethiopia have revealed several challenges, such as poor management and governance of human resource health and weak regulatory capacity for HRH [38,44]. The MOH has developed a 10-year HIS human resource development road map (2020-2030) and policy on eHealth architecture [45] to address these challenges. This road map provides guidance about the future need for human resources, ensuring proper national HIS support.

Regarding the “ICT infrastructure” domain, the DHIS2 implementation is currently at the repeatable stage (score=2.14). However, there are challenges such as unstandardized measurements of power outages, outdated infrastructure, and limited and unstable network and internet connectivity that hinder its effectiveness. Despite the objectives of the information revolution road map of the country to improve health care delivery through the appropriate use of ICTs, health care facilities in Ethiopia still face issues such as inadequate power sources, poor planning for replacing outdated and damaged equipment, and a lack of business continuity plan related to power supply [38,44,46]. Similar challenges have been reported in other African countries such as Kenya [47], Botswana [48], and Cameroon [49].

#### ICT Infrastructure

Establishing hardware and networking standards and guidelines for procurement would ensure the incorporation of related technological advancements without any hindrance to the interoperability of systems [50,51]. On the basis of the study findings, it is evident that the main determinants of DHIS2 information use are the availability of computers, communication networks, internet services, trained staff, and legislation [52]. In Africa, there is a need for an ample supply of computers, networks, internet services, and other accessories as well as training staff to boost ICT infrastructural prerequisites for the proper functioning of DHIS2 [53]. Particularly in Ethiopia, communication gaps between the internet service providers and health institutions, lack of follow-up and lack of technical support from the MOH and regional health bureaus, lack of regular network and internet connectivity assessment and reporting methods, and redundant internet or wide area network connection options are among the major challenges [44].

#### Standards and Interoperability

“Standards and interoperability” is another domain of DHIS2 implementation that is at the defined stage (score=3). Despite the availability of eHealth architecture that guides the interoperability of digital health systems, limited documentation has been found in Ethiopia outlining the standards for data exchange [45]. Health data exchange and harmonization rely on registry services, but in Ethiopia, there are limitations in the regular update and feedback process of the implemented core services. A client registry has not been developed, and Ethiopia does not have a national digital identification program [44]. Consistent with the call for developing standardized indicators by the World Health Assembly Resolution 63.16 [54], the Ethiopian MOH publishes health and health-related indicators annually [55], which is crucial for facilitating personal and aggregate data exchange.

#### Data Quality and Use

DHIS2 implementation has reached the defined stage (score=3) of maturity in terms of data quality and use. This means that there are regular data quality assessments and audits; quarterly dissemination of analytics and findings to the stakeholders; and automated data reporting using DHIS2, including sex-disaggregated data, where applicable, at all health facilities. However, there are still gaps in the national data quality governing body meeting conducted on an irregular basis to maintain data quality. Furthermore, there is a lack of common measurement metrics or indexes to monitor the progress of data or information use. Researchers agree that this lack of data quality and use is a major problem in LMICs [56,57] and needs to be addressed to solve the complex global health challenges. Thus, efforts to build a culture of data quality and use should be prioritized to ensure the effective use of data for improved health outcomes.

### Limitations

This assessment has some limitations. The assessment was conducted among participants with different levels of experience and knowledge regarding DHIS2 implementation in Ethiopia. Therefore, the rating might be affected by this variation. We have attempted to minimize this limitation by letting participants have more time to discuss their differences and reach consensus. We did not collect data about DHIS2 implementation at the health facility level due to resource limitations. Therefore, the actual implementation status might not be reflected. However, we have attempted to involve participants who closely support and monitor DHIS2 implementation at the facility level.

### Conclusions and Recommendations

The study used the SOCI maturity model toolkit to assess the maturity level of DHIS2 implementation in Ethiopia. The study found that DHIS2 implementation was at the defined stage of maturity in 4 of 5 domains, with a plan to move to the managed level (score=4.09) by 2025 by addressing the identified gaps.

The development of a standardized and basic DHIS2 process at the national level and the development of a 10-year strategic plan to guide the implementation of digital health systems, including DHIS2, were identified as strengths. However, the lack of workforce competency guidelines to support the implementation of DHIS2 and the gaps in knowledge and skills required to accomplish DHIS2 tasks at all levels of the health system were found to be the challenges in successfully implementing DHIS2.

The study also found that the country was in the emerging phase in terms of interoperability and data use components. Therefore, we recommend the following:

1. Clearly defined regulatory body, processes, and procedures to ensure compliance with DHIS2 standards, policies, and regulations should be established nationally. A process to review, validate, and enforce the implementation of policies, legislation, and regulations in DHIS2 should be regularized and updated as necessary to reflect the changes within the health domain. Metrics regarding DHIS2 compliance and noncompliance should be collected, recorded, and reported regularly.
2. A platform to review DHIS2 users’ competencies should be established to ensure continuous performance improvement and alignment with health care goals. This can be achieved through annual knowledge and skill evaluations and certification of the workforce. Planning, human capacity requirements, and availability related to digital health systems such as DHIS2 should be continuously improved based on the national digital health strategic plan.
3. Clear national plans and procedures for network management should be established. A dedicated network support team should be put in place at least at the district level. This will sustain the implementation of DHIS2 in the relevant facilities and offices at all levels. In addition, connectivity gaps should be documented and addressed as part of a standard process.
4. Integration of the DHIS2 data exchange and management process into the national HIS or health plan should be tracked, monitored, and reviewed through a standardized process. Moreover, it would be beneficial to have an interoperability laboratory for new exchange partners to test or for onboarding and to have a certification process.
5. Computerized alerts and reminders to managers, care providers, and patients; clinical guidelines; condition-specific order sets; focused patient data reports and summaries; documentation templates; diagnostic support; and contextually relevant reference information, among other tools, should be provided by integrating decision support apps into the DHIS2 system.

Overall, the recommendations aimed to improve the DHIS2 implementation in Ethiopia by addressing the identified gaps and enhancing the strengths. By implementing these recommendations, the country can improve the efficiency and effectiveness of its HIS, which can lead to better health outcomes for its population. However, the successful implementation of the recommendations depends on the prioritization and the capacity of the ministry and its stakeholders to allocate the necessary budget and resources.

## Appendix

Multimedia Appendix 1 Comparison of features of Information Systems for Health Maturity Assessment Toolkit and Stages of Continuous Improvement for maturity assessment and improvement planning purposes.

Multimedia Appendix 2 Maturity levels of each domain, component, and subcomponent for the District Health Information System version 2 implementation.

Multimedia Appendix 3 District Health Information System version 2 road map development for the leadership and governance domain.

Multimedia Appendix 4 District Health Information System version 2 road map development for the management and workforce domain.

Multimedia Appendix 5 District Health Information System version 2 road map development for the information and communication technology infrastructure domain.

Multimedia Appendix 6 District Health Information System version 2 road map development for the standard and guideline domain.

Multimedia Appendix 7 District Health Information System version 2 road map development for the data quality and use domain.

## Abbreviations

DHIS2: District Health Information System version 2
HIS: health information system
ICT: information and communication technology
IS4H: Information Systems for Health Maturity Assessment Toolkit
LMICs: low- and middle-income countries
MOH: Ministry of Health
SOCI: Stages of Continuous Improvement
USAID: United States Agency for International Development
WHO: World Health Organization
