# Supplementary material for: Maturity Assessment of District Health Information System Version 2 Implementation in Ethiopia: Current Status and Improvement Pathways
Source: JMIR Med Inform. 2024 Jul 26;12:e50375. doi: 10.2196/50375 (PMC11316158; doi:10.2196/50375)
Supplement: Multimedia Appendix 1 [file medinform_v12i1e50375_app1.docx]

Multimedia Appendix 1: Comparison of features of IS4H and SOCI for maturity assessment and improvement planning purposes

|  |  | Comparison of models | |
| --- | --- | --- | --- |
|  |  | Information  Systems for Health (IS4H) | HIS Stages of Continuous  Improvement  (SOCI) |
| Features of maturity  assessment model | Key features of the planned  assessment  (What is expected) |  |  |
| Scope  (General) | All components of a sub-system | Includes assessment of the four goals of PAHO’s Information Systems for Health Initiative at a national level (HIS in general) | Includes domains and sub-domains that can be aligned with a health system or HIS strategic plan (HIS in general) |
| Domains, sub-domains, and questions | Cover all major determinants of HIS subsystem performance | - IS4H includes several questions that are too specific to adapt to a subsystem - The subcomponents of SOCI can almost always be adapted to a specific subsystem - Consists 4 Domains, 26 components - 134 questions (Very detailed) | 5 domains, 13 associated components, and 39 subcomponents (Less detailed) |
| HIS components covered | All  components of HIS subsystem | 1. Data management and information 2. technologies 3. Management and governance 4. Knowledge management and sharing 5. Innovation | More domains of SOCI than IS4H are more applicable for specific HIS sub-systems   1. HIS leadership and governance 2. HIS management and workforce 3. HIS information and communication technologies (ICT) infrastructure 4. HIS standards and interoperability 5. HIS data quality and use |
| Relevance: fit for purpose | Facilitate maturity assessment and road mapping | A comprehensive tool guiding maturity assessment and strategic plan development | A comprehensive tool guiding maturity assessment and road mapping for continuous improvement |
| Validity,  reliability, and credibility of the model | Previously tested  Accepted by relevant stakeholders | - Developed and tested in Latin American countries - Need to consult stakeholders for credibility - Specific items quantitatively aggregated to measure each subcomponent make the model more reliable | - Developed and tested in LMICs - Need to consult stakeholders for credibility - Assessments are at a sub-component level and involve more subjectivity, thus yielding less reliable measures. |
| The precision of maturity measure | A precise enough scale to allow monitoring of progress | Level 5 mostly represents a defined state IS4H considers innovation as a separate domain while SOCI considers innovation and improvement as a characteristic of the highest level of performance under each HIS subcomponent | - Level 1 (Emerging) to - Level 5 (Optimized) - Level 5 mostly represents the presence of continuous improvement in the sub-system |
| Effort required to adapt the model for assessment of an HIS subsystem | A model that can be adapted within a short time and a small team | Several items are not applicable to a subsystem. Removing them would affect the metrics significantly. | Most items are defined at a higher level so that almost every item can be adapted to a specific HIS subsystem. However, the ‘patient identifier’ subcomponent is not applicable as DHIS2 is mostly for aggregate data exchange. |
| Feasibility of data collection | Doable through desk review and stakeholder consultation | Doable through desk review and stakeholder consultation | Doable through desk review and stakeholder consultation |
