# Supplementary material for: Maturity Assessment of District Health Information System Version 2 Implementation in Ethiopia: Current Status and Improvement Pathways
Source: JMIR Med Inform. 2024 Jul 26;12:e50375. doi: 10.2196/50375 (PMC11316158; doi:10.2196/50375)
Supplement: Multimedia Appendix 2 [file medinform_v12i1e50375_app2.docx]

[Multimedia Appendix 2: Maturity levels of each domain, component, and subcomponent for the DHIS2 implementation.](https://medinform.jmir.org/api/download?filename=e2eb60b7b6336eec8932be4cf3715557.docx&alt_name=50375-855812-1-SP.docx)

| **Domains and Components** | **Subcomponents** | **Current Status** | **Target Status** |
| --- | --- | --- | --- |
| Overall maturity Level |  | 2.81 | 4.09 |
| I. Leadership and governance |  | 2.83 | 3.83 |
| A. Strategy |  | 3.5 | 4.5 |
|  | 1. HIS strategic planning | 4 | 5 |
|  | 2. Monitoring and evaluation (M&E) plan | 3 | 4 |
| B. Policy, legal, and regulatory framework, and compliance |  | 2 | 3 |
|  | 1. Existence of HIS policies and legislation | 2 | 3 |
|  | 2. Policy compliance enforcement | 2 | 3 |
| C. Leadership and governance organizational structures and functions |  | 3 | 4 |
|  | 1. HIS leadership and coordination | 3 | 4 |
|  | 2. HIS organizational structure and functions | 3 | 4 |
| II. Management and workforce |  | 2.9 | 4.08 |
| A. Workforce capacity and development |  | 2.2 | 3.67 |
|  | 1. HIS competencies (knowledge, skills, and abilities) | 2 | 4 |
|  | 2. HIS training and education (includes continual professional development) | 3 | 4 |
|  | 3. HR policy | 1.67 | 3 |
| B. Financial management |  | 3.6 | 4.5 |
|  | 1. HIS financing plan | 4 | 5 |
|  | 2. Resource mobilization | 3.33 | 4 |
| III. Information and communication technologies (ICT) infrastructure |  | 2.14 | 3.89 |
| A. Operations and maintenance |  | 2.44 | 3.67 |
|  | 1. Reliable power/electricity | 2 | 3 |
|  | 2. ICT business Infrastructure support | 2.33 | 4 |
|  | 3. Hardware | 3 | 4 |
| B. Communication network (LAN and WAN) |  | 2 | 4 |
|  | 1. Networks and Internet connectivity | 2 | 4 |
| C. Business continuity |  | 2 | 4 |
|  | 1. Business continuity processes and policies | 2 | 4 |
| IV. Standards and interoperability |  | 3.19 | 4.61 |
| A. Standards and guidelines |  | 3.33 | 4.33 |
|  | 1. HIS standard guidelines | 4 | 5 |
|  | 2. Data set definitions (clinical, laboratory, commodities, and indicator) | 4 | 5 |
|  | 3. Data and exchange standards | 2 | 3 |
| B. Core services |  | 3.25 | 5 |
|  | 1. Master facility list | 3 | 5 |
|  | 2. Indicator registry | 3 | 5 |
|  | 3. Terminology management | 3 | 5 |
|  | 4. Unique person identity management | N/A | N/A |
|  | 5. Enterprise architecture | 4 | 5 |
| C. Interoperability (data exchange) |  | 3 | 4.5 |
|  | 1. Person data exchange | N/A | N/A |
|  | 2. Aggregate data exchange | 4 | 5 |
|  | 3. Commodity management data exchange | N/A | N/A |
|  | 4. Data exchange security | 2 | 4 |
| V. Data quality and use |  | 3 | 4.06 |
| A. Data quality assurance |  | 3 | 4 |
|  | 1. Data quality assurance and quality control | 3 | 4 |
|  | 2. Data management | 3 | 4 |
| B. Data use |  | 3 | 4.11 |
|  | 1. Data use availability strategy | 4 | 5 |
|  | 2. Information/data availability | 3 | 4 |
|  | 3. Data use competencies | 1 | 3 |
|  | 4. User/stakeholder engagement | 4 | 5 |
|  | 5. Data synthesis and communication | 4 | 5 |
|  | 6. Reporting and analytics features | 3 | 4 |
|  | 7. Data use impact | 2 | 3 |
|  | 8. Data collection alignment with workflow | 4 | 5 |
|  | 9. Decision support (clinical or other) | 2 | 3 |
