# Supplementary material for: Maturity Assessment of District Health Information System Version 2 Implementation in Ethiopia: Current Status and Improvement Pathways
Source: JMIR Med Inform. 2024 Jul 26;12:e50375. doi: 10.2196/50375 (PMC11316158; doi:10.2196/50375)
Supplement: Multimedia Appendix 3 [file medinform_v12i1e50375_app3.docx]

Multimedia Appendix 3: DHIS2 roadmap development for the leadership and governance domain

| Domain and sub-component | Gaps to be addressed | Activity |
| --- | --- | --- |
| Leadership and Governance |  |  |
| Strategy |  |  |
| HIS strategic planning | - The system is not readily responsive & a continuous improvement process is not maintained. - lack of continuously updated plans and enforcements for DHIS2 strategy - lack of continuous requirement analysis - Lack of SOP at the local level - Version difference between global and local system - Customization is needed based on the country’s standards | - A continuous improvement planning process shall be maintained - Implement digital health planning with budget and resources to customize it to international standards - Develop and promote an accountability and transparency framework with clearly defined roles and responsibilities - Strengthen/Establish a designated body responsible for taking corrective measures - Strengthen key stakeholders/partners collaboration - Design a strategy for additional fund & resource mobilization for DHIS2 initiatives - Conducting global landscape analysis - Establish and make functional and local DHIS2 continuous improvement team /community of practice - Push with HISP Oslo to get the Ethiopian Calendar in the DHIS2 core - Revisit and improve the DHIS2 implementation strategy in light of continuous changes |
| Monitoring and evaluation (M&E) plan | - There is no clearly defined and standardized monitoring and evaluation tool for DHIS-2 implementation. - No separate and independent institution which assesses the implementation. - The M & E platform is not regular, & does not fully capture the impact at the point of service delivery. - There is no standardized tool for continuous (M&E), updating the available tool is required | - Promote a transparent M&E system by creating a public health information access platform (Portal, Forum). - Ensure the structure is in line with HIS strategy - Intensify standardized tools with comprehensive implementation - Conduct consistent data collection for evaluation - Develop/review indicators with health care service and HIS strategy - Engagement and stakeholders: advance private involvement and agencies, NGOs - Conduct impact analysis: periodic and regular such as mid-term evaluation with advanced data analysis - Checking whether it is with the strategic directions - Formulating a team to assess the implementation status of different HIS Priority - Conduct consistent data collection for evaluation |
| Policy, legal, and regulatory framework and compliance |  |  |
| Existence of HIS policies and legislation | - There are limited regulatory frameworks & legislations to guide DHIS2 implementation. - It is not comprehensive & there is no established team to follow the status & enforce the regulations. | - Develop a comprehensive capacity-building and mentorship strategy - Strengthen/Establish a designated/responsive body responsible for checking the timely finalization, endorsement, and implementation of HIS policies and legislation - #Strengthen key stakeholders/partners collaboration forum (including private sectors, academia, Civil society organizations, professional associations...) - Develop, finalize, and endorse HIS legislation, regulation, and policies to guide DHIS2 implementation. - Establish Knowledge sharing and advocacy team and apply Innovative methods - Implement and monitor core policies and regulations. |
| Policy compliance enforcement | - There are no established enforcement mechanisms initiated so far. - There is no defined body /structure to enforce & follow the compliance of the limited existing policies | - Design organizational structure and create a defined/responsible body with roles and responsibilities, processes, and procedures to ensure compliance with SOPs. - Create a structure for correction/remediation. - Define standard measures or metrics of compliances (collected, recorded, reported, and verified) - Develop accountability framework such as data quality |
| Leadership and governance organizational structures and functions |  |  |
| HIS leadership and coordination | - Lack of infrastructure and capacity for ensuring leadership and coordination. - The national leadership, monitoring and evaluation, and resources available to organize and coordinate DHIS2 are not consistent throughout the healthcare system. | - Establish a follow-up mechanism/platform to strengthen coordination of the DHIS2 initiative - Stakeholder engagement shall be applied across all sub-national levels. - Design and implement the process of DHIS2 initiatives at all levels in the health sector and in line with the national DHIS2 and M&E plan - Maximize stakeholder coordination and use efficiently in terms of resource - Monitor ongoing reviews by the national-level coordination group of HIS activities, - Communications for continuous improvement to meet changing HIS strategy and/or health goals. - Capacity building training, benchmark. DSS i.e. Dashboard for leadership - Avail DHIS2 data with public portals - Track the implementation of DHIS2 via different high-level forums |
| HIS organizational structure and functions | - DHIS2 Implementation Core Team is not specific to TOR - No approved structure for HITs working on DHIS2 at facility levels. Moreover, there is no written Job description provided to HITs that guides their duties on data to day-to-day activities on DHIS2 functions. - There is no defined career progress & retention mechanism - Unsatisfactory job evaluation grading | - Create a standardized and formal process for review and updates of organizational structure with JDs for HIS across all national and sub-national levels. - Prepare and execute a national plan for career development training and retention for each HIS personnel. - Develop capacity building and mentorship program/plan for continuous professional and career development. - Work on career training and retention for each job series/cadre. - Diversified training (short- and long-term training) including advanced analytics - Design career training such as health informatics specialization - Add Creating a national plan for career developments - Conducting assessments regarding career developments, push mechanisms, HIT satisfaction, Evaluating the load, payment, and retention mechanism |
