# Supplementary material for: Maturity Assessment of District Health Information System Version 2 Implementation in Ethiopia: Current Status and Improvement Pathways
Source: JMIR Med Inform. 2024 Jul 26;12:e50375. doi: 10.2196/50375 (PMC11316158; doi:10.2196/50375)
Supplement: Multimedia Appendix 4 [file medinform_v12i1e50375_app4.docx]

[Multimedia Appendix 4: DHIS2 roadmap development for the management and workforce domain](https://medinform.jmir.org/api/download?filename=9f9c7bee8865f4111ffc7f08872fc730.docx&alt_name=50375-855817-1-SP.docx)

| Domain and sub-component | Gaps to be addressed | Activity |
| --- | --- | --- |
|  |  |  |
| Workforce capacity and development |  |  |
| HIS competencies (knowledge, skills, and abilities) | - There is no established and disseminated national organizational chart with clear descriptions of duties and responsibilities for each DHIS2 workforce - DHIS2 workforce capability assessments and analyses are not conducted - Unfair Job Evaluation Grading (JEG) done for HIT/HI required positions at all levels | - Develop well-defined roles and responsibilities for HIS workforce at the national level and disseminate them to the sub-national level - Develop an assessment checklist that measures HIS competencies (if any conduct tool validation) at the national level - Job evaluation re-grading for HIT or HI/IT required positions at all levels - Revise HIS workforce structure at all levels |
| HIS training and education (include continual professional development) | - The trainings are irregular, not need-based and inclusive for all workforce - No sufficient infrastructure to support different modes of DHIS2 training, including distance learning. - Fragmented team for HIS workforce competencies development | - Expand and strengthen the community of practice team at the national level overseen by designated government authority (DHIS 2 academy) - Revise educational program curricula/Strengthen pushing factors for curriculum update - Incorporate Pre-service or in-service training in Continuous professional development (CPD) - Establish a national DHIS2 workforce team, stakeholder engagement |
| HR policy | - Numbers are not sufficient to meet DHIS2 workforce needs at the national level | - Job evaluation re-grading for HIT or HI required positions at all level - Apply motivation and retention mechanisms. - Assess HIS workforce which includes availability and job requirements |
| Financial management |  |  |
| HIS financing plan | - An established financial management system is not fully owned, reviewed, tracked, and revised by the government. | - Strengthen recurrent budget allocation and manage overall the budget for DHIS - Strengthen Public-private - Monitoring and evaluation of financial management - Aligned with various sources of finance - Institutionalize resource mobilization |
| Resource mobilization | - The resource mobilization plan for DHIS2 activities is not integrated into the health plan at the appropriate level of implementation at all level - The review process is not standardized and not regularly reviewed | - Strengthen resource mobilization for DHIS2 at the sub-national level and frequent audit - Validate DHIS resource mobilization/mapping tool and disseminate at all levels |
