# Supplementary material for: Maturity Assessment of District Health Information System Version 2 Implementation in Ethiopia: Current Status and Improvement Pathways
Source: JMIR Med Inform. 2024 Jul 26;12:e50375. doi: 10.2196/50375 (PMC11316158; doi:10.2196/50375)
Supplement: Multimedia Appendix 5 [file medinform_v12i1e50375_app5.docx]

Multimedia Appendix 5: DHIS2 roadmap development for the Information and communication technologies (ICT) infrastructure domain

| Domain and sub-component | Gaps to be addressed | Activity |
| --- | --- | --- |
| ICT Infrastructure |  |  |
| Operations and maintenance |  |  |
| Reliable power/electricity | - There are no standardized metrics for measuring and follow-up of power outage - No continuous electricity supply - No Standardized metrics for measuring power outages and the duration of outages are defined | - Establish power availability metrics and conduct electrification assessment including frequency of failure - Design and propose alternate power and power redundancy solutions including Solar, generator, UPSs, ACs, etc in collaboration with the Health Infrastructure CEO and other stakeholders establishing (MOU) |
| ICT business Infrastructure support | - Lack of human power or capacity for maintenance - Lack of support - Absence of standards for maintenance | - Assessment of the ICT infrastructure, IT audit, and putting the system in place (digital health resource management tool) for monitoring availability and functionality and implementation of the standard - Prepare and enforce support strategy, including SLA, strengthen helpdesk at least at Admin offices, supportive supervision at national and sub-national levels, operationalize ICT policy - Capacity building(maintenance) plan and enforcement - Deployment plan, enhancing the main data center, operationalizing the DR site, exploring the alternative hosting mechanisms, Service providers, local and international hosting companies |
| Hardware | - The sub-national capacity is not adequate in terms of hardware. - ICT capacity to support ICT equipment installation and maintenance | - Enhance the hardware distribution via mobilizing resources in collaboration with stakeholders at the sub-national level. Enforce the facilities to maintain their budget with MOU - Update and Operationalize hardware specification, evaluation, and inspection mechanisms in line with system requirements - Assessment of hardware and capacity of personnel - regular training on the hardware at all levels based on assessment |
| Communication network (LAN and WAN) |  |  |
| Networks and Internet connectivity | - The network (LAN and WAN) connectivity is not adequate | - Enhance coverage and speed of Internet and network via assessment of including Health net and other sources (Internet, WordNet, satellite) and with stakeholders (including ISP) - Establish and upgrade the LAN and WAN coverage at facilities including a plan in collaboration with stakeholders including PPP groups |
| Business continuity |  |  |
| Business continuity processes and policies | - Inadequate Business continuity and processes and policies and plans and not enforced | - Strengthen or update and enforce Business continuity plan (Backup procedures, Disaster recovery procedures, Personnel, IT inventory, Disaster recovery sites, Restoration procedures) - Operationalize the DR site - Incorporate Business continuity-related policy within ICT policy and establish enforcing mechanisms |
