# Supplementary material for: Maturity Assessment of District Health Information System Version 2 Implementation in Ethiopia: Current Status and Improvement Pathways
Source: JMIR Med Inform. 2024 Jul 26;12:e50375. doi: 10.2196/50375 (PMC11316158; doi:10.2196/50375)
Supplement: Multimedia Appendix 6 [file medinform_v12i1e50375_app6.docx]

Multimedia Appendix 6: DHIS2 roadmap development DHIS2 standard and guideline domain

| Domain and sub-component | Gaps to be addressed | Activity |
| --- | --- | --- |
| Standards and Interoperability |  |  |
| Standards and guidelines |  |  |
| HIS standard guidelines | - lack of continuously improving existing DHIS2 standard guidelines and adopting new standards | - Design and implement a process framework/workflow that helps to improve and adopt new guidelines |
| Data set definitions | - DHIS2 should use a centralized system that can subscribe and publish to the data indicator list | - Improve dhis2 capability/workflow to receive/send indicator list to a centralized indicator registry - Develop a guideline/standard on how to exchange indicator lists between DHIS2 and the centralized indicator registry - Establish a DHIS2 team to actively support and follow up the data exchange |
| Data and exchange standards | - Unable to certify data exchange standards - No responsible body for certification and updating data exchange standards | - Design a mechanism to approve and certify dhis2 data exchange standards - Enhance the interoperability lab that is responsible for standardizing, testing, approval, and certifying data exchange standards |
| HIS Core services |  |  |
| Master facility list | - Lack of communication or feedback process to review facility list data exchange between MFL and DHIS2 | - Establish a team to follow up the data exchange process and provide continuous feedback - Design metrics to evaluate the data exchange progress and provide review/ update for the data exchange middleware /IOL team #provide capacity building training to sustain the data exchange |
| Indicator registry | - Absence of workflow to receive indicator list from external indicator registry - Lack of quality assurance framework to evaluate and update indicator list | - Enhance dhis2 capability to accept indicator list from an external system (OCL) - Establish a team to evaluate and maintain the indicator list captured on the indicator registry system |
| Terminology management | - Absence of workflow to receive indicator list from external indicator registry - Lack of quality assurance framework to evaluate and update indicator list | - Enhance dhis2 capability to accept terminology lists from an external system - Establish a team to evaluate and maintain the terminology list registered on the terminology management system |
| Enterprise architecture | - dhis2 business, application, information/data, and technology capabilities are not included in the enterprise architecture | - align dhis2 capabilities in the national business, application, information/data, and technology capabilities in the enterprise architecture |
| Interoperability (data exchange) |  |  |
| Aggregate data exchange | - Absence of aggregate data exchange that includes minimum data exchange between DHIS2 and other systems and submitted to the certification organization | - Evaluate the DHIS2 readiness to accept aggregate data from multiple data sources and design a guideline on how to send/receive aggregate data exchange - Finalize the aggregate data exchange standard document and send it to the certification organization |
| Data exchange security | - No security requirements to maintain DHIS2 data exchange with other systems | - Establish a data exchange security mechanism to ensure standard-based data exchange between dhis2 and other HIS |
