# Supplementary material for: Maturity Assessment of District Health Information System Version 2 Implementation in Ethiopia: Current Status and Improvement Pathways
Source: JMIR Med Inform. 2024 Jul 26;12:e50375. doi: 10.2196/50375 (PMC11316158; doi:10.2196/50375)
Supplement: Multimedia Appendix 7 [file medinform_v12i1e50375_app7.docx]

Multimedia Appendix 7: DHIS2 roadmap development for the DHIS2 data quality and use domain

| Domain and sub-component | Gaps to be addressed | Activity |
| --- | --- | --- |
| Data Quality and Use |  |  |
| Data quality assurance |  |  |
| Data quality assurance and quality control | - DHIS-2 data quality audits are done semi-manually - Regular review of data Quality Assurance plan not done - Information use TWG didn't meet regularly to plan and act on data quality issues regularly - Data exchange from other systems is manual and triggers data quality issues | - Automate data quality audits by strengthening validation rules and other features like built-in analytics and data quality apps - Conduct regular reviews of data quality assurance plans and practice at all levels - Strengthen/Revitalize Information Use TWGs at all levels - Automate aggregate data exchange between DHIS-2 and other systems like EMR - Strengthen the use of trend Analysis at a lower level during data entry - Ensure accountability for compliance in data quality at the individual, health facility, and administrative levels. |
| Data management | - Supportive Supervisions are conducted irregularly - DHIS2 data management SOP isn't reviewed regularly - Regular data quality monitoring lacks consistency across the health system | - Review and update data quality monitoring SOPs, Guidelines, and Feedback mechanisms regularly at all levels - Strengthen Data Management training across all levels - Conduct standardized and regular Supportive Supervision - Automate Supportive Supervision tools for feedback and action points tracking. - Strengthen Mentorship activities in health institutions. |
| Data use |  |  |
| Data use availability strategy | - DHIS2 has not been updated regularly to meet the evolving decision-making needs of program managers, policymakers, and providers interacting with HIS | - Update and implement data use strategies on a regular basis to meet the changing data use needs of care providers and program managers - Ensure a long-term health plan to foster a data-driven culture. |
| Information/data availability | - Data availability is not monitored for continuous improvement or to meet emerging health sector needs; not all stakeholders have access to the data they need - DHIS2 does not effectively support the secondary use of data. | - Make available, monitor for continuous improvement or to meet emerging health sector needs, and ensure data accessibility for all stakeholders - Integrate and manage a data warehouse to ensure that data from secondary data sources is available to users. |
| Data use competencies | - A knowledge management roadmap should be in place - DHIS2 data use competencies are not evaluated regularly. | - Review and standardize expected data use competencies for DHIS2 data use at National and Sub National Levels like academy level data quality, advanced data analytics - Conduct Competencies assessment at National and Sub National Levels - Establish/ Strengthen routine and continuous competency assessment platforms. - Provide Training based on the assessment findings. /Ensure availability of updated knowledge management roadmap - Initiate/Strengthen/ continuous Professional Development (CPD) |
| User/stakeholder engagement | - The guidance for stakeholder engagement is not reviewed and updated regularly to address emerging and future user decision-making needs. | - Conduct stakeholder analysis periodically - Revise, endorse, and implement health harmonization and alignment manual (HHM) - Prepare and enforce SOPs for stakeholder engagement |
| Data synthesis and communication | - Guidance on the design and use of information products is not reviewed and revised regularly | - Provide capacity-building training on advanced data management techniques such as data mining/data science, Machine Learning, interactive data visualization tools, and data triangulation - Strengthen the system of data analysis, synthesis, and communication |
| Reporting and analytics features | - Lack of real-time data use across all facilities and levels. - Limited advancement features to support data analysis and visualization. | - Ensure all government health facilities use DHIS2 online - Enhance validation rules and other data quality, analysis, and visualization features in DHIS2 platforms. - Integrate DHIS2 with other digital Platforms like POWER-BI - Conduct continuous system improvement by adding new features |
| Data use impact | - Parameters for evaluating the impact of data use are not well integrated into HIS and/or health plans - They are not up to date, monitored, or reviewed by a designated governing body | - Ensure the availability of measurement metrics, and strengthen planning, monitoring, and evaluation - Strengthen Impact evaluation studies using the routine data. |
| Data collection alignment with workflow | - The alignment of DHIS2 data collection processes with existing workflows is not regularly evaluated for continuous improvement. | - Establish/enhance regular review mechanisms for data collection alignment with existing workflow. - Consider giving data entry access to service providers in DHIS-2. - Implement automated data exchange between DHIS-2 and other systems. |
| Decision support (clinical or other) | - Inadequate decision-making tools that incorporate program guidelines and documentation templates - DHIS2 does not include knowledge-based decision-support systems. | - Integrate knowledge management concepts in the DHIS2 pre-and in-service training - Ensure a functional coordination mechanism to use decision and KM effectively for informed decision-making - Conduct regular end users' need assessment for Decision Support features in DHIS2. - Develop/Customize automated decision support features for Data quality, completeness, and use. |
